# A framework for organizing and selecting quantitative approaches for benefit-harm assessment

**DOI:** 10.1186/1471-2288-12-173

**Published:** 2012-11-19

**Authors:** Milo A Puhan, Sonal Singh, Carlos O Weiss, Ravi Varadhan, Cynthia M Boyd

**Affiliations:** 1 Johns Hopkins Bloomberg School of Public Health, 615 North Wolfe Street, Room E6153, Baltimore, MD, 21205, USA; 2 Division of General Internal Medicine, Johns Hopkins School of Medicine, Baltimore, USA; 3 Geriatric Medicine and Gerontology, Michigan State University, Grand Rapids, USA; 4 Division of Geriatrics, Johns Hopkins School of Medicine, Baltimore, USA

## Abstract

**Background:**

Several quantitative approaches for benefit-harm assessment of health care interventions exist but it is unclear how the approaches differ. Our aim was to review existing quantitative approaches for benefit-harm assessment and to develop an organizing framework that clarifies differences and aids selection of quantitative approaches for a particular benefit-harm assessment.

**Methods:**

We performed a review of the literature to identify quantitative approaches for benefit-harm assessment. Our team, consisting of clinicians, epidemiologists, and statisticians, discussed the approaches and identified their key characteristics. We developed a framework that helps investigators select quantitative approaches for benefit-harm assessment that are appropriate for a particular decisionmaking context.

**Results:**

Our framework for selecting quantitative approaches requires a concise definition of the treatment comparison and population of interest, identification of key benefit and harm outcomes, and determination of the need for a measure that puts all outcomes on a single scale (which we call a benefit and harm comparison metric). We identified 16 quantitative approaches for benefit-harm assessment. These approaches can be categorized into those that consider single or multiple key benefit and harm outcomes, and those that use a benefit-harm comparison metric or not. Most approaches use aggregate data and can be used in the context of single studies or systematic reviews. Although the majority of approaches provides a benefit and harm comparison metric, only four approaches provide measures of uncertainty around the benefit and harm comparison metric (such as a 95 percent confidence interval). None of the approaches considers the actual joint distribution of benefit and harm outcomes, but one approach considers competing risks when calculating profile-specific event rates. Nine approaches explicitly allow incorporating patient preferences.

**Conclusion:**

The choice of quantitative approaches depends on the specific question and goal of the benefit-harm assessment as well as on the nature and availability of data. In some situations, investigators may identify only one appropriate approach. In situations where the question and available data justify more than one approach, investigators may want to use multiple approaches and compare the consistency of results. When more evidence on relative advantages of approaches accumulates from such comparisons, it will be possible to make more specific recommendations on the choice of approaches.

## Background

Some decisions on health care interventions are straightforward because the benefits clearly outweigh the harms or vice versa. Many decisions, however, require careful balancing of the benefits and harms. For example, in order to decide on the use of aspirin for the prevention of myocardial infarction, one would typically consider the risk reduction for myocardial infarction over a certain period of time (e.g. 10 years) as well as the increased risks for hemorrhagic stroke and gastrointestinal bleeding [1]. One would also need to consider that the benefit-harm comparison varies across patients since the risks for these outcomes and absolute treatment effects depend much on an individual patient’s profile including her or his preferences. Sometimes, the decisionmaking context is even more complex than for aspirin. Tamoxifen for the prevention of breast cancer, for example, modifies the risk not only for breast cancer, but also for endometrial carcinoma, bone fractures, pulmonary embolism, stroke and cataracts [2]. As with aspirin, the benefit-harm comparison of tamoxifen depends on a woman’s profile, which in this case includes age, the risk for invasive breast cancer, race and whether the uterus is intact or has been removed [3].

In situations where multiple outcomes, patient profiles and also patient preferences need to be considered, it is challenging to compare benefits and harms without a quantitative approach. Without a quantitative approach, it is not verifiable if and how different outcomes, patient profiles, patient preferences, and sources of evidence were considered and weighted. Non-quantitative assessments of benefits and harms may lead to inappropriate decisions for or against treatments. This lack of transparency may be less problematic in an individual decisionmaking context, but it seems unacceptable when major regulatory decisions or clinical guideline recommendations are at stake. A number of quantitative approaches have been developed and applied to handle the multidimensionality of a benefit-harm assessment [3-8]. But there is little guidance on the selection of an appropriate quantitative approach for a particular clinical question in a benefit and harm assessment [5,7,9]. Past reviews of quantitative approaches for benefit and harm assessment have not organized methods according to important characteristics [5,7,9]. A framework that recognizes their important characteristics and organizes them accordingly could be a step forward to understanding the common and different elements of existing approaches, to guide their further development, and support investigators, who plan to conduct a quantitative benefit and harm assessment, in their choice of approach.

Such an organizing framework could be particularly attractive for organizations or investigators who conduct or use systematic reviews because the literature is mostly silent about the use of quantitative benefit and harm assessment approaches in the context of a systematic review. Familiarity with available approaches and their key characteristics will help systematic reviewers to develop protocols that specify all sources and types of data needed for benefit-harm assessments. For example, additional database searches may be needed to identify evidence on baseline risks, harms or patient preferences that would be missed by standard searches that commonly focus on randomized trials. Therefore, our aim was to review existing quantitative approaches for benefit-harm assessment and to develop an organizing framework that helps understanding of differences among methods and their selection for a specific clinical question.

## Methods

### Scope of the literature review

Our purpose was to review quantitative approaches for benefit and harm assessments that use formulas or graphical displays to compare the benefits and harms. We evaluated methods feasible for use in systematic reviews or to use the data synthesized in systematic reviews. We did not consider theoretical frameworks and qualitative approaches for benefit and risk assessment, nor approaches that have not been used in the medical field. It is important to note that our review focused on quantitative assessment and not on the entire process of a benefit-harm assessment (e.g. in the context of a new drug approval) that includes quantitative and qualitative processes [10,11]. Also, we did not review approaches for making treatment recommendations for populations or individual patients because this requires consideration of specific health care contexts, costs of treatment, and other contextual factors, which is beyond the scope of most systematic reviews.

### Review of the literature

We began our search for quantitative benefit and harm approaches with key articles culled from the investigators’ reference libraries, including prior work on approaches for assessing benefits and harms. We looked for articles that were written for quantitative benefit and harm assessment, which included consideration of at least one outcome for both benefit and harm of a medical or public intervention. We included approaches that analyzed benefit and harm outcomes entirely separately as well as approaches that provided a benefit and harm comparison metric where single or multiple benefit and harm outcomes are put on the same scale, which we will call the benefit-harm comparison metric (e.g. Quality-adjusted Life Years [QALYs] or probability scale). We screened the reference lists of all included articles for more relevant articles. The group then discussed each article that seemed potentially relevant, as described below. We also reviewed the manuals of the Evidence Based Practice Center program of the Agency for Healthcare Research and Quality (AHRQ) (http://www.ahrq.gov/clinic/epc/) and of the Cochrane Collaboration.

We did not perform a formal systematic review of the literature because a review on the topic of quantitative benefit and harm assessment already existed [5] and because our focus was on organizing available approaches. We capitalized on the work done to create a list of relevant approaches [5], which allowed us to devote adequate resources to the main focus of developing an organizing framework that helps understanding the differences among methods and their selection for a specific clinical question.

### Defining the decisionmaking context of a benefit and harm assessment and identification of key characteristics of existing quantitative approaches for benefit and harm assessment

Our team, consisting of clinicians, epidemiologists and statisticians, discussed the identified quantitative approaches for benefit and harm assessment and the context in which they might be used in 12 one-hour sessions. The discussion served to define properties that characterize quantitative approaches for benefit and harm assessments. Based on these characteristics we developed a simple algorithm that may guide investigators, systematic reviewers, guideline developers or policymakers in their selection of a quantitative approach for benefit-harm assessment. We iteratively defined key characteristics with which existing quantitative approaches for benefit and harm assessment can be described and that allow comparisons across quantitative approaches. We also recorded limitations inherent to each of them that may threaten their usefulness or limit their applicability for certain questions of benefit and harm. In addition, we prepared examples for quantitative approaches for benefit-harm assessment that would highlight differences between them.

## Results

### Decisionmaking context of benefit and harm assessment

We identified three characteristics of a decisionmaking context that need to be defined. First, the treatment comparison and population for which the benefit-harm assessment is made should be characterized. The comparison of interest can be an intervention versus no intervention or an intervention A versus an intervention B. A population can be broadly defined, for example as a screening or primary prevention population, or be restricted to a particular setting (e.g. primary care) or a particular clinical population (e.g. patients with manifest coronary heart disease). Secondly, the key benefit and harm outcomes of interest for which the evidence and the benefit-harm comparison is sought should be identified. The figure shows that there might be a single benefit and a single harm outcome that are of interest, or there could be multiple outcomes. As is noted in literature on the more general processes of assessing the benefits and harms of interventions in guidelines and systematic reviews, patient-important outcomes might be preferred as key outcomes but, sometimes, a surrogate outcome may be considered if there is a strong correlation with patient-important outcomes or if no evidence on patient-important outcomes is available. Finally, one should decide whether a benefit-harm comparison metric is desired that puts all outcomes on a common scale so that a single number will inform about the comparison of benefits and harms. The decisionmaking context is likely to be important to decide on the need for a benefit-harm comparison metric because the output of the available quantitative approaches differ substantially and may not be appropriate for all decisionmakers such as patients and their health care providers, clinical or public health guideline developers, regulatory agencies, policymakers, or payers.

### Overview of existing quantitative approaches for benefit-harm assessment

**Figure 1:**
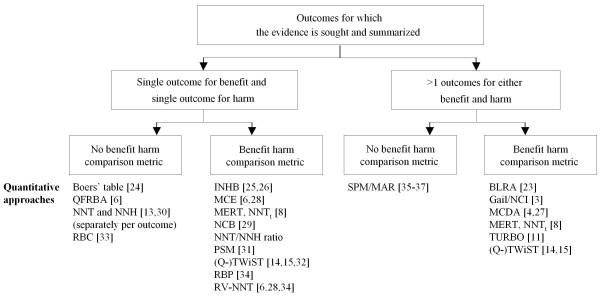
**Key characteristics of the benefit-harm question that may guide selection of quantitative assessment for benefit-harm assessment.** Abbreviations: *INHB*, Incremental net health benefit; *MCE*, Minimum clinical efficacy; *NCB*, Net Clinical Benefit; *NNT*, Number needed to treat; *NNH*, Number needed to treat for harm; *Q-Twist*, (Quality-adjusted) Time without Symptoms and Toxicity: *RBC*, Risk–benefit contour; *RV-NNT*, Relative value adjusted number needed to treat; *QFRBA*, Quantitative Framework for Risk and Benefit Assessment; *TURBO*, Transparent Uniform Risk Benefit; *BLRA*, Benefit-less-risk analysis; *PSM*, Probabilistic simulation methods; *MERT*, Minimum Target Event Risk for Treatment; *MCDA*, Multicriteria decision analysis: *RBP*, Risk–benefit plane; *SPM*, Stated preference method; *MAR*, Maximum acceptable risk.

We identified 16 approaches, which can be grouped into two broad categories (Figure 1): One category comprises simpler approaches that typically deal with a single outcome for benefit (e.g. prevention of myocardial infarction) and one outcome for harm (e.g. gastrointestinal bleeding). The single benefit and harm can, however, also be composite outcomes (e.g. cardiovascular events) that summarize several outcomes (acute coronary syndrome, stroke, and cardiovascular death). The more complex approaches consider multiple outcomes for either benefit or harm, or both. Of note, although some approaches like the Number needed to treat (NNT) [12,13] and Number needed to harm (NNH) and (Quality-adjusted) Time without Symptoms and Toxicity (Q- TWiST) [14,15] are mostly used when there is a single benefit and a single harm outcome. Researchers can use these approaches separately for different outcomes in situations where multiple outcomes are important (e.g. multiple NNTs and NNHs). In the figure, we categorized approaches according to how they are typically used in the medical literature (e.g. NNT and NNH are typically used for single outcomes), but listed a few of them in two categories if we could not clearly categorize them (e.g. Minimum Target Event Risk for Treatment [MERT], (Quality-adjusted) Time without Symptoms and Toxicity).

The figure shows that the use of a benefit and harm comparison metric further distinguishes various approaches. The number of outcomes, the need for a benefit and harm comparison metric, and the quality and quantitative of available data will likely drive the selection of approaches.

To facilitate the understanding of and between the approaches, we selected three approaches for a more detailed description (Table 1). NNT and NNH are examples of an approach that can be used if a single benefit and a single harm outcome is of interest or when multiple outcomes are treated separately. Multicriteria decision analysis (MCDA) [16,17] and the Gail/National Cancer Institute [3] approach provide examples for approaches where multiple outcomes are considered and put on a benefit-harm comparison metric.

**Table 1:** Examples for quantitative approaches for benefit-harm assessment

	
**NNT and NNH**	**Examples where researchers consider single benefit and harm outcomes with or without a benefit-harm comparison metric**
	NNT and its harm outcome counterpart, NNH, are the approaches researchers most widely use to measure risk and benefit reported in systematic reviews and evidence-based medicine [12,13]. Also, NNT is the metric clinical practice guidelines most commonly use to address benefit-harm comparisons. NNT or NNH are the number of individuals who need to be treated over a specified period of time for one person to benefit or be harmed, respectively, and vary as the specified treatment time varies. Studies mostly present NNT and NNH separately (i.e. they are not combined on a benefit-harm comparison metric such as the ratio of NNT and NNH). For example, the Based Clinical Practice Guidelines on Antithrombotic Therapy in Atrial Fibrillation of the American College of Chest Physicians present NNTs based on a systematic review of randomized trials of oral anticoagulant therapy versus no antithrombotic therapy: “The efficacy of warfarin was consistent across studies with an overall relative risk reduction of 68 percent (95 percent confidence interval [CI], 50 to 79 percent) analyzed by intention-to-treat [18]. The absolute risk reduction implies that 31 ischemic strokes will be prevented each year for every 1,000 patients treated (or 32 patients needed to treat for 1 year to prevent one stroke, NNT = 32)”.In contrast, studies scarcely use the NNT/NNH ratio. One reason for the rare application of this benefit-harm comparison metric may be that investigators or guideline developers are reluctant to weigh benefit and harm outcomes equally on the same scale because of uncertainty about their relative clinical importance. To address this dilemma, Guyatt et al. proposed using relative value units to weight the NNT or NNH [19]. An example using the NNT/NNH ratio is a review of trials of an antidepressant drug which summarized benefit defined as response and remission of depression and harm as suicide [20].
**Multicriteria decision analysis**	**Example where multiple benefit and harm outcomes and preferences are considered**
	The Analytic Hierarchy Process A is a commonly used approach for MCDA studies is. We illustrate this approach using the comparative effectiveness review of oral hypoglycemic agents for type 2 diabetes. The first step in Analytic Hierarchy Process analysis consists of defining the goal of the decision, the alternatives being considered, and the criteria that determines how well patients and clinicians can expect that alternatives will meet the goal [4,21]. The criteria are organized into a hierarchical decision model with a desired goal of determining the best treatment of type 2 diabetes at the top; the alternatives thiazolidinediones, metformin, and sulfonylurea’s at the bottom; and the criteria in between**.** Operationally, we could define two criteria as being necessary for determining the best treatment: 1) its ability to maximize benefits via glucose reduction, and 2) its ability to minimize harms or medication-related adverse effects. Researchers could divide the criteria on maximizing benefits into three sub-criteria: health-related quality of life, microvascular benefit (such as improvements in incidence of neuropathy, nephropathy and diabetic retinopathy), and potential macrovascular benefit. Researchers could subdivide the criteria on minimizing risk of harm into six sub-criteria based on medication-related adverse events: congestive heart failure, fractures in women, macular edema, bladder cancer, myocardial infarction, and hypoglycemia. In the second step, researchers obtain information about how well the alternatives can be expected to fulfill the decision criteria from a systematic review. The third step consists of two parts: 1) comparing the ability of the alternative treatments to fulfill the prespecified criteria (maximizes benefit and minimizes harm) using standard Analytic Hierarchy Process pairwise comparisons, and 2) assessing the importance of these criteria to the decision goal. In the fourth step, researchers can combine the scales created in step 3 to create a summary score (the benefit-harm comparison metric) indicating how well the alternative treatments can be expected to meet the decision goal [21]. The fifth step consists of performing sensitivity analyses to explore the effects of changing the estimates or judgments used in the original analysis. The main advantage of MCDA is that it is that identifies the extent to which every criterion, judgment, and weight contributes to the benefit-harm comparison metric and that it also incorporates uncertainty. Additional visual representation of the results allow one to gain understanding and articulate the divergence between relevant stakeholders [4].
**Gail/National Cancer Institute**	**Example where multiple benefit and harm outcomes are considered with a benefit-harm comparison metric**
	Some decisionmaking contexts are more complicated because there are many potential treatment outcomes as well as sources of uncertainty. A well known example of a very challenging decision is whether or not to use of tamoxifen to prevent breast cancer. Tamoxifen reduces the risk for invasive and in situ breast cancer substantially and prevents some bone fractures [2]. On the other hand, it increases the risk for endometrial cancer, stroke, and pulmonary embolism. The National Cancer Institute under the leadership of Gail developed an approach to deal with multiple outcomes [3]. Rather than simplifying the benefit-harm assessment to single outcomes, as many investigators and guideline developers do, they estimated the probability of various outcomes for women with and without tamoxifen therapy over a period of 5 years. Based on observational studies, surveillance registries or placebo arms of randomized trials, they first estimated the expected number of invasive breast cancers, in situ breast cancers, hip fractures, endometrial cancers, strokes, pulmonary embolisms, deep vein thromboses, colles’ fractures, spine fractures, and cataracts each per 10,000 women, over 5 years, in the absence of tamoxifen treatment. They estimated these numbers overall and stratified for different age and race categories. Then, based on the Breast Cancer Prevention Trial, they estimated, for each outcome, the expected number of the same outcomes but with tamoxifen treatment. Again this was per 10,000 women over 5 years, as well as overall, and it was stratified for different age and race categories. They also took competing risk from death into consideration. In order to put all outcomes on the same scale but to also consider the relative clinical importance of these outcomes, they categorized the outcomes into life-threatening, severe and other outcomes and suggested weighting them with some factor (e.g. 1 for life-threatening, 0.5 for severe and 0.0 for other outcomes). These categories and weights can be modified according to patient or treatment provider preferences. Ultimately, the results of the benefit-harm assessment are presented as the net number of events prevented or in excess per 10,000 women treated with tamoxifen over a period of 5 years. For example, for a 45-year-old woman with a uterus and a 4 percent risk of invasive breast cancer over 5 years, the net number of events prevented (weighted by their clinical importance) is 196 per 10,000 women with this profile (the expected number of prevented invasive and in situ breast cancers was 299/10,000 woman but there were 59 women/10,000 woman with harm such as endometrial cancer, stroke, pulmonary embolism, or deep vein thrombosis). The net benefit (benefit minus harm events) varied considerably and was positive for some profiles (as example above) but negative for others (e.g. black woman with age 50–59 years and a 5-year risk of invasive breast cancer of 4 percent).

### Description of key characteristics of 16 quantitative approaches for benefit-harm assessment

We focus here on additional key characteristics that enable researchers to understand the similarities and differences and choose the appropriate approaches for their benefit-harm question and context. Papers focusing on each individual approach have described in detail the 16 approaches although not in the context of systematic reviews [5].

We identified the following additional key characteristics:

1. *The type of data needed:* Individual patient data have advantages over aggregate data typically available for evidence synthesis. In an individual study, information on the co-occurrence of benefit and harm outcomes is available for each patient. An important consequence of the availability of such individual data is that researchers can consider the joint probability of benefit and harm outcomes.

2. *The type of analyses:* Analyses for benefit-harm assessment could include any type of statistical analysis. There is a major distinction, however, between approaches that are data driven (deterministic) and approaches that use modeling (stochastic) where data are not described by unique values, but rather by probability distributions.

3. *The type of benefit-harm comparison metric:* Researchers may use absolute and relative metrics as well as QALYs.

4. *Assumptions*: In benefit-harm analyses researchers need to make a number of assumptions. For example, if researchers use a benefit-harm comparison metric, the assumption is that it is justifiable that outcomes are combined on a single scale. Other assumptions relate to the joint occurrence of separate outcomes. Some approaches assume that separate outcomes occur independently, which may be justifiable in some instances.

5. *Consideration and incorporation of patient preferences:* Some quantitative approaches explicitly consider patient preferences for different outcomes in order to weight the benefits and harms.

6. *Types of presenting benefit risk comparisons:* Researchers can use various formats to present the results of a quantitative benefit-harm assessment. Researchers may present the benefit-harm comparison as a difference in the number of events between a treatment or no treatment, or as a ratio. They may also express the comparison by the time gained or lost without symptoms through a treatment. Or, researchers can use graphics that depict the benefit-harm comparison for patients at different outcome risks or based on other patient characteristics.

In Table 2, we compare each of the 16 approaches against these key characteristics. Table 3 provides a more detailed discussion of each approach. Three approaches require individual patient data (Table 2) whereas 13 approaches do not require individual patient data. This means that, although most quantitative approaches were developed for use in randomized trials and observational studies, researchers can also use most of these approaches at the synthesis stage (systematic reviews). Fourteen of the approaches are data driven but four of them may also use simulation. One approach (Probabilistic Simulation Methods [PSM]) is entirely based on simulation. Twelve of the 16 approaches put benefit and harm outcomes on the same scale to provide a benefit and harm comparison metric. Only four approaches provide measures of uncertainty around the benefit and harm comparison metric. Four approaches could consider the joint distribution of benefit and harm outcomes for the estimation of uncertainty. But many examples using these four approaches do not consider the dependence between benefit and harm, but only consider their marginal distributions (i.e. consider them to be independent,). Five approaches use composites outcome for benefit and composite outcomes for harm while 12 approaches use multiple outcomes. Researchers have adapted or potentially could adapt nine of the 16 approaches to incorporate patient preferences.

**Table 2:** Key characteristics of quantitative approaches for benefits and harm assessment

**Approaches for B&H assessment**	**BLRA**[22]	**Boers**[23]	**Gail**[3]	**INHB**[24,25]	**MCDA**[4,26]	**MCE**[6,27]	**NCB**[28]	**NNT& NNH**[12,13]	**PSM**[29]	**QFRBA**[6]	**Q-TWiST**[14,15,30]	**RBC**[31]	**RBP**[32]	**RV-NNT**[6,27]	**SPM & MAR**[33-35]	**TURBO**[11]	**Number of approaches in each category**
**Key characteristics**																	
Types of data																	
Require individual patient data	Yes	Yes	No	No	No	No	No	No	No	No	No	No	No	No	Yes	No	Indiv: 3
																	No: 13
Types of analyses																	
Data driven (DD) versus simulation (S)	DD	NA	DD/S	DD	DD	DD	DD/S	DD	S	DD	DD	DD/S	DD	DD	DD	DD	DD: 14
																	S: 4
Types of B&H metrics																	
Absolute versus relative measures versus QALY versus other (O)	Other	A	A	QALY	A / relative	A	A	A	A	A / relative	A /QALY	A	A	A	A	A / relative	Absolute: 14
																	Relative: 3
																	QALY: 2
Assumptions																	
Put B&H outcomes on same scale	yes	no	yes	yes	yes	yes	yes	yes	yes	no	yes	no	yes	yes	no	yes	Yes: 12
																	No:4
Uncertainty estimates for B&H assessment	no	no	no	no	yes	no	yes	no*	yes	NA	no	yes	no	no	NA	no	Yes: 4
																	No: 12
Joint distribution of B&H outcomes considered for uncertainty estimates	no	NA.	P	NA	no	NA	no	no	P	NA	NA	P	P	no	NA	NA	Possible: 4
																	No: 4
																	n.a.: 8
Multiple endpoints versus composite outcomes for B&H	M	Comp	M	M	M	M	M	M/ Comp	M	M	M	M	Comp	M	M	Comp	Multiple:12
																	Comp: 5
Consideration of preferences																	
Explicitly considers preferences for B&H assessment:	yes	no	yes	no	yes	both	yes	no	both	no	yes	no	no	yes	yes	no	Yes: 9
																	No:9
Types of presenting benefit risk comparison																	
B&H difference versus B&H ratio versus Time gained/lost versus B&H graphic versus other	D	Graphic	D	D	D, ratio, other	D	D	Ratio	D	D, ratio, other	Time, D	Graphic	Graphic	Ratio	D	Graphic	Difference: 10
																	Ratio: 4
																	Time: 1
																	Graphic: 3

^#^ NNT and NNH are most commonly used separately but put on the same scale if their ratio is taken.

*NA*, Not applicable; *BLRA*, Benefit-less-risk analysis; *B&H*, Benefit and harm assessment; *INHB*, Incremental net health benefit; *MCDA*, Multicriteria decision analysis; *MCE*, Minimum clinical efficacy; *NCB*, Net Clinical Benefit; *NNT*, Number needed to treat; *NNH*, Number needed to treat for harm; *PSM*, Probabilistic simulation methods; *QFRBA*, Quantitative Framework for Risk and Benefit Assessment; *Q-Twist*, (Quality-adjusted) Time without Symptoms and Toxicity; *RBC*, Risk–benefit contour; *RBP*, Risk–benefit plane; *RV-NNT*, Relative value adjusted number needed to treat; *SPM*, Stated preference method; *MAR*, Maximum acceptable risk; *TURBO*, Transparent Uniform Risk Benefit Overview; *P*, Possible; *D*, Difference; *A*, Absolute; *M*, Multiple.

**Table 3:** Brief description of the 16 approaches for quantitative benefit-harm assessment

	
**Benefit-less-risk analysis (BLRA)**	Researchers developed benefit-less-risk analysis, which combines benefit and harm into a single metric, primarily for clinical trials [36]. This analysis takes advantage of individual patient data. For each patient, researchers record the benefit (yes or no) and express the harm as a value between 0 and 1. Researchers present the relationship between benefit and risk as risk subtracted from benefit, which allows for statistical testing of comparisons between treatment groups. Patient preferences expressing the relative importance of benefit and harm outcomes can be considered. Benefit-less-risk analysis is a method that takes advantage of individual patient data. Thus, if researchers applied this method in a systematic review, they would need to gather individual patient data from the primary studies.
**Boers’ 3x3 table**	This quantitative approach does not require any statistical models but suggests a way of organizing outcome data on the same scale [23]. Researchers need individual patient data. They split the outcomes of patients into three categories and display the number of patients with a certain benefit-harm profile (e.g. major benefit and minimal harm) in a 3x3 table. Researchers do not consider treatment effects directly since they construct separate 3x3 tables for each treatment group. As a consequence, no measures of uncertainty are available. Researchers do not consider patient preferences, but instead the clinicians’ view or agreement of what constitutes minimal, moderate, or major benefit or harm. The method is both feasible for single trials or systematic reviews. A disadvantage is that, although each table is simple and easy to read, it requires readers to somehow estimate treatment effects across tables or to provide a benefit and harm comparison metric, and thus challenges rather than facilitates conclusions concerning benefits and harms.
**Gail/National Cancer Institute**	This is one of the most comprehensive approaches for benefit and harm assessment and considers various data sources to balance the benefits and harms of a treatment [3]. As described above, researchers can calculate the benefit and harm comparison metric as the sum of benefit and harm outcome rates per patient profile. They can incorporate patient preferences by looking only at one severity grade, or by putting weights on outcome rates that reflect patient perception of events: very severe, severe, or moderately severe. The approach does not consider the joint distribution of benefit and harm outcomes but could potentially be extended to do so. However, by looking at benefit and harm comparison estimates across patient profiles, one gets an impression of how the net benefit changes, even qualitatively, as the baseline risk changes. This approach is resource intensive because it considers multiple data sources and multiple outcomes. The United States Preventive Services Taskforce used a similar though simplified approach to make recommendations on the use of aspirin for the prevention of myocardial infarction [1]. Similar to the tamoxifen example, researchers estimated the number of benefit (myocardial infarction) and harm (bleedings) events per 1,000 men or women based on observational data and the evidence on treatment benefits and combined harms with these outcome estimates. The benefit-harm comparison metric provided the number of net events (benefit minus harm) prevented or in excess when aspirin is used [1].
**Incremental net health benefit**	Incremental net health benefit provides a benefit and harm comparison metric, using QALYs to place one or more benefits and harms on the same scale, and calculates the difference between benefit and harm between treatments (thus a result >0 is favorable) [24,25]. A key requirement for this approach is the valid measurement of utilities or the sometimes inaccurate transformation of quality of life scores into utilities. Also, it is often difficult to distinguish between the effects of benefits and harms when using utilities.
**Multicriteria decision analysis (MCDA)**	A multi-criteria decision analysis allows for a systematic decisionmaking in complex situations involving tradeoffs, by considering various harms and benefits associated with treatments [16,17]. Researchers develop a decision tree model to incorporate benefits from clinical trials and harms such as adverse effects. It allows for input from various stakeholders who may assign different preference weights to the risks and benefits. MCDA represents an approach to reduce the multidimensionality of benefit-harm assessment in a systematic way and makes judgments explicit and transparent. It allows for decisionmaking in the presence of uncertainty and can incorporate data from multiple sources including systematic reviews. The challenges of its application to systematic reviews include getting reliable information on various preferences, agreement on all relevant important benefits and harms and the relative importance and weighting of these outcomes, and the need to specify a decision context since systematic reviews are usually conducted to meet the needs of multiple decisionmakers. The flexibility of MCDA also poses challenges for benefit-harm assessment as systematic reviews are unable to inform on all inputs, especially less tangible inputs (e.g. societal values, opportunity costs) that may alter harm and benefit balance in a particular decision context.
**Minimum clinical efficacy**	Minimum clinical efficacy incorporates harm and benefit into a benefit and harm comparison metric. The benefit is the difference in efficacy and harm, both of which are expressed on a probability scale by applying relative risks reductions (treatment benefits) and increases (harms) to absolute probabilities as observed in untreated groups [6,27]. Researchers consider the intervention as having minimal clinically efficacy if the difference between benefit and harm is positive or above a minimally acceptably threshold. Minimum clinical efficacy can consider relative utilities. A limitation includes the inability to provide uncertainty estimates for the benefit and harm comparison metric.
**Net clinical benefit**	Similar to the Gail/National Cancer Institute approach, the calculation of the net clinical benefit considers different data sources such as randomized trials, observational studies, and patient preferences, and provides profile-specific benefit-harm comparison estimates [28]. Researchers calculate the benefit-harm comparison metric as the sum of all expected benefits minus the sum of all expected harms. They calculate the benefit from the pooled relative risk reductions (based on meta-analysis) that they apply to patients at different risk for the benefit outcome (e.g. stroke). They calculate the expected harm from the risks for the harm outcome, and the patient preferences for the harm outcome. They calculate net clinical benefit using a Bayesian approach where they model all steps simultaneously (meta-analysis, calculation of expected benefit, and expected harm). A major advantage of this approach is its flexibility to combine different data sources and place distributions on each parameter. Thereby researchers can qualify uncertainty around the parameters. Net clinical benefit considers patient preferences for different outcomes, but similar to other approaches, the selection of particular values for preferences has a large impact on the net clinical benefit estimates. In the Figure, we categorized the approach as considering only single benefit and harm outcomes because published applications of the approach considered only one benefit and one harm outcome. But the approach offers, theoretically, enough flexibility to consider multiple outcomes.
**Number needed To treat (NNT) And number needed To treat To harm (NNH)**	The (NNT) NNH) refer to the number of individuals who need to be treated over a specified period of time with the intervention for one person to benefit and experience the harm [12,13].NNT and NNH depend on baseline risk (and are thus sensitive to different patient profiles) and the degree of relative risk reduction provided by the intervention (which researchers often assume is constant across the disease spectrum, but in fact may actually vary). Researchers can calculate NNT and NNH for single outcomes (e.g. NNT for exacerbations vs. NNH for fractures) or for composite outcomes for both benefit and harm. But since the concept of NNT is one of frequency and not of importance, researchers should only calculate the NNT and NNH ratios or differences for outcomes of similar importance [37]. When researchers calculate a ratio or difference between NNT and NNH as a benefit and harm comparison metric, researchers assume their independence and may need to extrapolate so that the ratios refer to the same time period. Researchers cannot calculate NNT and NNH for continuous outcomes unless such outcomes are dichotomized. NNT and NNH is perhaps the most widely used measure of risk and benefit reported in systematic reviews and evidence-based medicine. Extensions of the NNT/NNH ratio approach include: the threshold NNT, the minimum target event risk for treatment (MERT), and the subject-year adjusted NNT [8]. The threshold NNT reflects the point at which the risks and costs of a clinical intervention balance the benefit, and the minimum target event risk for treatment defines the minimum target event risk at which the intervention is justified. Subject-year adjusted NNT uses subject years as the denominator instead of participants, to better account for time-on-treatment for participants. For example, if there are two events per 1,000 subject years in the control group and one event per 1,000 subject years in the intervention group, the NNT is 1,000 subject years. This means that with treatment, one fewer event would occur with every 1,000 subject-years. Methods for providing uncertainty for these benefit and harm comparison metrics are available [8]. The NNT/NNH, threshold NNT and MERT all seem feasible within a systematic review context.
**Probabilistic simulation methods (PSM)**	The PSM use probabilistic simulations for benefit and harm comparison estimates using Monte Carlo methods. PSM can incorporate parameters from multiple data sources (systematic reviews of randomized clinical trials and observational studies), patient preferences (e.g. from conjoint analysis) and different patient profiles [7,29,32].This method estimates uncertainty around the benefit and harm comparison estimate, with or without consideration of the joint distribution of benefits and harms (depending on the availability of individual-level data or reporting of covariances).
**Quantitative framework for risk and benefit assessment (QFRBA)**	QFRBA reports on benefit and harm separately. It does not provide a benefit and harm comparison metric and uncertainty estimates for benefit or harm outcomes are only available for the separate treatment effects [5]. An advantage of this method is that keeps benefit and harm separate, leaves room for incorporation of preferences by decisionmakers and consideration of multiple outcomes. Also, QFRBA is probably the way most systematic reviews currently report or discuss the benefit and harm assessment.
**(Quality-adjusted) time without symptoms and toxicity (TWiST or Q-TWiST)**	TWiST compares treatments in terms of the time without symptoms gained versus the time lost due to the experience of adverse effects [14,15]. It therefor puts the benefit and harm on the same scale (time). Q-TWiST is a further development where time is converted into QALYs [30]. Here the benefit and harm comparison metric is the difference between the drug associated gain in QALYs and the loss in QALYs associated with the treatment due to adverse effects. Numerous oncology studies have used Q-TwiST. The major advantage of this method is the ability to incorporate patient preferences, which may change over time. The method depends heavily on the availability of measurements that allow estimating the length of time periods without symptoms and of time periods where adverse effects are experienced. Also, measurement instruments need to be highly specific so that a distinction between benefit and harm is possible. For example, quality of life and some preference-based instruments often provide a composite score that already synthesizes the overall experience of a patient. Also, QALYs itself values health states rather than changes in health states, and lack of a measure of uncertainty around these measurements may limit the usefulness of this method. In a systematic review, this method may be difficult to apply since QALYs associated with benefit and harm are unlikely to be reported in reports of primary studies.
**Risk–benefit Contour (RBC)**	The risk–benefit contour plot is a graphical method to assess benefits and harms [31]. It portrays the probability of benefit for a new treatment compared to another treatment against the probability of harm for that new treatment (again as compared to another treatment). Contour lines portray the shape of this relationship for a number of different probabilities and confidence levels. The risk-benefit contour plot is a way to express uncertainty associated with certain pairs of benefit and harm. The plot conveys study-level relationships, and does not consider the dependence of the probability of benefit and harm at the individual level. Though the method does not incorporate weights (representing patient preferences) for each type of outcome, researchers could adapt it to do so. Researchers should probably view risk–benefit contour as a way to present data and visualize uncertainty. This way researchers can base the underlying analyses that yield the probability estimates on different statistical approaches such as various forms of Probabilistic Simulation Methods (PSM).
**Risk–benefit plane (RBP) and risk–benefit acceptability threshold (RBAT)**	The RBP and RBAT display in a simple figure, both separate estimates of benefit and harm and a benefit and harm comparison metric [29,32]. This method does not consider the individual-level dependence between benefit and harm. Using an absolute scale, this method plots the probability of benefit (from a comparison between two treatments) against the probability of harm. With this method, researchers refer to the slope created by a line between the origin and the two-dimensional result as the risk-benefit acceptability threshold. This method does not consider outcome weights that would reflect patient preferences.
**Relative value adjusted number needed To treat (RV-NNT)**	The major advantage of RV –NNT over NNT and NNH is that it allows for incorporation of preferences into the assessment of benefit and harm [6,27]. Otherwise it offers the same advantages as the NNT/NNH ratio approach, and suffers from some the same limitations. Systematic reviews would need information on preferences to incorporate this method.
**Stated preference method and maximum acceptable risk**	Researchers use SPM and MAR are used to survey patients on how much burden from adverse effects, or serious adverse events, they are willing to accept in order to experience the benefits of treatment [33,35,38,39]. Researchers need individual patient data for these approaches. The typical method to elicit preferences is discrete choice or conjoint analysis, where respondents have to pick their preferred treatment from two treatment scenarios that characterize the benefit and harm of these treatments. These approaches assume that the attractiveness of a particular treatment is a function of the benefit and harm attributes, which are combined in various ways in the different vignettes of the survey [34].
**Transparent uniform risk benefit overview (TURBO)**	The TURBO diagram displays the factors R and B. R is the sum of the most serious adverse effect (scored from 1–5) and the second most serious adverse effect (scored from 1–2) [11]. Researchers base the scores on the frequency and severity of the harm outcome. Similarly, factor “B” is the sum of the primary benefit (1–5), and the ancillary benefit (1–2) and researchers base the scores on the probability and extent of the benefit outcome. The T score represents the benefit and harm comparison metric and ranges from 1 (high R and low B score) to 7 (high B and low R score).Researchers typically use the TURBO diagram in a regulatory context (e.g. European Medicines Agency) and therefore, they base them on single trials, but researchers can also base them on systematic reviews. Researchers can base the factors R and B on absolute or relative measures of treatment effects for which uncertainty estimates are available. But there is no uncertainty estimates for the benefit and harm comparison metric (i.e. the “T” score). Unlike other approaches, the TURBO diagram explicitly considers not only one but two outcomes for both benefit and harm that are weighted differently (up to 2 or 5 points). Challenges to applying the TURBO method include arbitrary selection of the two benefit and harm outcomes from a comprehensive list of outcomes and the way researchers assign scores (combining frequency and importance of outcomes).

## Discussion

The main finding of our review of quantitative approaches for benefit and harm assessment used in the medical literature is a simple algorithm that categorizes existing quantitative approaches broadly into approaches that consider single or multiple benefit and harm outcomes and into approaches that use a benefit-harm comparison metric or present outcomes side by side. We also found that for most approaches, researchers use aggregate data so as to make the approaches suitable for systematic reviews even if that is not their intended purpose. Interestingly, only few approaches provide measures of uncertainty and none of the approaches considers a potential correlation between benefit and harm outcomes (joint distribution).

We identified a number of assumptions that researchers make when applying some of the quantitative approaches: First, for some approaches researchers assume that one or more benefit and harm outcomes can be put on the same scale to calculate a benefit and harm comparison metric. Challenges for putting different outcomes on the same scale include their relative importance to decisionmakers, simplification of the outcomes (e.g. dichotomizing continuous outcomes, which may lead to substantial loss of information), or different methods and timing in the ascertainment of different outcomes.

However, the advantages of a benefit-harm comparison metric may be substantial, for example, in the context of complex situations where multiple outcomes are important and where patient, provider, and policymaker preferences vary [7]. It is a great cognitive challenge to process such a multidimensional task without a benefit-harm comparison metric. The major advantage of using a benefit-harm comparison metric (over using an approach without such a common metric) is that it can make explicit assumptions about the relative importance of outcomes or the arbitrary selection of the evidence on benefits and harms or on baseline risks, and that sensitivity analyses can provide evidence as to how the benefit-harm comparison changes if different assumptions are made. Also, a single number may provide some advantages for the communication of benefit-harm comparison to patients because it avoids overwhelming the patients with data on multiple different outcomes.

Second, we were surprised to see that there were no quantitative approaches that considered or even discussed the joint distribution of benefit and harm outcomes, even when individual patient data were available. The joint distribution describes the correlation between benefit and harm outcomes. Trial reports commonly describe standard errors and confidence intervals for the benefit and harm outcomes separately, but rarely describe the joint distribution of the effects of the treatment on the benefit and harm outcomes. Without the joint distribution of all the effects, we have to assume independence of the benefit and harm effects. This may not yield a valid estimate of the uncertainty of the benefit-harm balance metric. Changes in reporting practices, such as online journal appendix materials or online repositories of covariance data for later data synthesis, could address this limitation. Systematic reviewers should keep in mind the limitation of not considering the joint distribution when interpreting results from a quantitative benefit and harm assessment.

The figure and table show a number of characteristics that help distinguish various existing quantitative approaches. While these characteristics are important for the selection of an appropriate quantitative approach, there are a number of additional considerations that researchers need to make because they have implications regarding the type of evidence included in the benefit-harm assessment. For example, clinical trials are commonly designed to provide high-quality evidence and sufficient power for benefit outcomes. Harms often receive much less attention in terms of accurate and valid methods of measurement [40]. Such asymmetry in the quality of outcome ascertainment affects the validity of a quantitative benefit and harm assessment, but it is yet unclear how to downgrade the quality of evidence for this reason.

In contrast to the framework developed here that focused entirely on the quantitative assessments, Lynd and others developed criteria that apply to the entire process of a benefit-harm assessment. This usually requires that researchers consider both quantitative and qualitative approaches to make conclusions regarding benefit-harm comparisons of health care interventions [10,11,41,42]. Lynd and others proposed 10 criteria for benefit-harm assessments--be universal, inclusive, comprehensive, patient-sensitive, easily interpreted consider preferences, define when benefits outweigh harms, incorporate uncertainty, be flexible and integrate economic evaluations) [41]. We agree with these guiding principles but also think that researchers cannot readily use them to judge the adequacy of specific quantitative approaches. Whether or not a specific approach is adequate depends much on the type and quality of available data. Regulatory decisionmakers, guideline developers, or users of the evidence are likely to perceive the ease of use and ease of interpretation of quantitative approaches very differently because of different levels of methodological expertise or different perspectives. Therefore, we believe that our framework for organizing quantitative approaches is complementary to, rather than competing with, what Lynd and others have proposed. The frameworks proposed by our team, Lynd, and others support a systematic, well-structured, and transparent process for reducing the multidimensionality of a benefit-harm assessment.

Our review showed that current quantitative approaches for benefit-harm assessment might need some further development. Firstly, many quantitative approaches identified here focus on binary outcomes that occur just once, with or without consideration of time to event. Current methods need extensions that also consider different types of data. Some patient-important outcomes, such as quality of life or symptoms, cannot be expressed appropriately as binary outcomes without substantial loss of information. Some benefit and harm events can occur several times so that the number of events per person-time needs to be considered rather than the proportion of persons with at least one event. Secondly, uncertainty estimates for the benefit and harm comparison metric (e.g. 95 percent confidence or credible intervals) are likely to be of key importance for decisionmakers and organizations making treatment recommendations. Researchers do not commonly report estimates of uncertainty that arises from sampling variability. In addition, none of the methods considers the joint distribution of benefit and harm outcomes. Researchers should develop statistical methods for considering joint distributions when estimating standard errors for benefit-harm comparison metrics. For systematic reviews it would be valuable to develop approaches for making assumptions about joint distributions because covariance matrices are rarely available from reports of primary studies and it may be challenging to request them from authors of primary studies. Thirdly, researchers should develop systematic approaches for sensitivity analyses that assess the influence of the various assumptions commonly made. One approach would be to agree on a list of standard sensitivity analyses for key aspects of a benefit-harm assessment. For example, data for estimating baseline risks (e.g. probability of outcome without treatment) can come from different sources (e.g. surveillance data, observational studies, and placebo arms of randomized trials). The best available evidence on treatment effects may sometimes come from single randomized trial or observational study rather than from meta-analyses. Researchers may be able to derive patient preferences by different eliciting techniques. A systematic outline of these options (the choices for the primary analysis and for sensitivity analyses), would make benefit-harm assessments transparent and give users of the evidence a sense for how sensitive the results are to different assumptions.

A strength of our review is the collaborative effort of clinicians, epidemiologists, and statisticians that helped us to develop a comprehensive framework for characterizing quantitative approaches for benefit-harm assessment. Some may perceive it as a limitation that we did not conduct a separate formal systematic review but we capitalized on an existing, recent review [5]. Also, we used an iterative approach of developing a framework rather than following a more standardized approach, such as Delphi-like procedures, to identify important characteristics of quantitative approaches for benefit-harm assessment. However, a more standardized approach also has its limitations because it does not allow discussing intertwined issues or considering different perspectives of an interdisciplinary research group in great depth.

We developed a framework for the use of quantitative approaches for benefit-harm assessment that can help researchers select specific approaches. We do not make recommendations for or against specific approaches. It is too early to make such recommendations because of the lack of evidence from studies that directly compare quantitative approaches applied to a specific question. The adequacy of approaches depends on the specific benefit-harm question and on the amount and quality of data that determine how justifiable certain assumptions are. In some situations, there may be a single approach that appears to be most appropriate. But commonly, there will be several approaches that are reasonable options given the question, the goal of the benefit-harm assessment, and the available data. In such situations, we suggest that investigators use several approaches, as commonly used in other areas [43,44], which acknowledges that none of them is perfect and based on some assumptions. The confidence in the results of benefit-harm assessments then depends on the extent to which different approaches arrive at similar results, and how useful they are to end-users. Evidence from studies applying multiple approaches to the same benefit-harm question, together with recognition of their advantages and disadvantages, would make it possible to identify approaches that are consistently superior over others, and to develop recommendations for specific approaches.

## Competing interests

The authors declare that they have no competing interests.

## Authors’ contribution

All authors read and approved the final manuscript.

## Funding

This work was funded by a contract to AHRQ, Rockville, MD (Contract No. HHSA 290-2007-10061-I). The findings and conclusions in this document are those of the author(s), who are responsible for its content, and do not necessarily represent the views of AHRQ. No statement in this report should be construed as an official position of AHRQ or of the U.S. Department of Health and Human Services.

Dr. Boyd was supported the Paul Beeson Career Development Award Program (NIA K23 AG032910, AFAR, The John A. Hartford Foundation, The Atlantic Philanthropies, The Starr Foundation and an anonymous donor).

## Pre-publication history

The pre-publication history for this paper can be accessed here:

http://www.biomedcentral.com/1471-2288/12/173/prepub

## References

[B1] Aspirin for the prevention of cardiovascular disease U.S. Preventive Services Task Force recommendation statement Ann Intern Med 2009 150 6 396 404 19293072 10.7326/0003-4819-150-6-200903170-00008

[B2] Fisher B Costantino JP Wickerham DL Redmond CK Kavanah M Cronin WM Vogel V Robidoux A Dimitrov N Atkins J Tamoxifen for prevention of breast cancer: report of the National Surgical Adjuvant Breast and Bowel Project P-1 Study J Natl Cancer Inst 1998 90 18 1371 1388 10.1093/jnci/90.18.1371 9747868

[B3] Gail MH Costantino JP Bryant J Croyle R Freedman L Helzlsouer K Vogel V Weighing the risks and benefits of tamoxifen treatment for preventing breast cancer J Natl Cancer Inst 1999 91 21 1829 1846 10.1093/jnci/91.21.1829 10547390

[B4] Dolan JG Multi-criteria clinical decision support: A primer on the use of multiple criteria decision making methods to promote evidence-based, patient-centered healthcare Patient 2010 3 4 229 248 10.2165/11539470-000000000-00000 21394218 PMC3049911

[B5] Guo JJ Pandey S Doyle J Bian B Lis Y Raisch DW A review of quantitative risk-benefit methodologies for assessing drug safety and efficacy-report of the ISPOR risk-benefit management working group Value Health 2010 13 5 657 666 10.1111/j.1524-4733.2010.00725.x 20412543

[B6] Holden WL Juhaeri J Dai W Benefit-risk analysis: a proposal using quantitative methods Pharmacoepidemiol Drug Saf 2003 12 7 611 616 10.1002/pds.887 14558185

[B7] Lynd LD Najafzadeh M Colley L Byrne MF Willan AR Sculpher MJ Johnson FR Hauber AB Using the incremental net benefit framework for quantitative benefit-risk analysis in regulatory decision-making–a case study of alosetron in irritable bowel syndrome Value Health 2010 13 4 411 417 10.1111/j.1524-4733.2009.00595.x 19744297

[B8] Walter SD Sinclair JC Uncertainty in the minimum event risk to justify treatment was evaluated J Clin Epidemiol 2009 62 8 816 824 10.1016/j.jclinepi.2008.09.017 19216053

[B9] O'Neill RT A Perspective on Characterizing Benefits and Risks Derived From Clinical Trials: Can We Do More? Drug Inf J 2008 42 3 235 245 10.1177/009286150804200305

[B10] European Medicine Agency Reflection paper on benefit-risk assessment methods in the context of the evaluation of marketing authorisation applications of medicinal products for human use 2008 Doc. Ref. EMEA/CHMP/15404/2007 http://www.ema.europa.eu/docs/en_GB/document_library/Regulatory_and_procedural_guideline/2010/01/WC500069634.pdf, last accessed February 16 2012

[B11] European Medicine Agency Report of the Committee for Medicinal Products Working Group on Benefit-Risk Assessment Models and Methods 2007 Doc. Ref. EMEA/CHMP/15404/2007 http://www.ema.europa.eu/docs/en_GB/document_library/Regulatory_and_procedural_guideline/2010/01/WC500069668.pdf, last accessed February 16 2012

[B12] Laupacis A Sackett DL Roberts RS An assessment of clinically useful measures of the consequences of treatment N Engl J Med 1988 318 26 1728 1733 10.1056/NEJM198806303182605 3374545

[B13] Cook RJ Sackett DL The number needed to treat: a clinically useful measure of treatment effect BMJ 1995 310 6977 452 454 10.1136/bmj.310.6977.452 7873954 PMC2548824

[B14] Gelber RD Goldhirsch A Cole BF Evaluation of effectiveness: Q-TWiST. The International Breast Cancer Study Group Cancer Treat Rev 1993 19 Suppl A 73 84 7679323 10.1016/0305-7372(93)90060-5

[B15] Gelber RD Goldhirsch A Cole BF Wieand HS Schroeder G Krook JE A quality-adjusted time without symptoms or toxicity (Q-TWiST) analysis of adjuvant radiation therapy and chemotherapy for resectable rectal cancer J Natl Cancer Inst 1996 88 15 1039 1045 10.1093/jnci/88.15.1039 8683634

[B16] Mussen F Salek S Walker S A quantitative approach to benefit-risk assessment of medicines - part 1: the development of a new model using multi-criteria decision analysis Pharmacoepidemiol Drug Saf 2007 16 Suppl 1 S2 S15 17546573 10.1002/pds.1435

[B17] Mussen F Salek S Walker S Phillips L A quantitative approach to benefit-risk assessment of medicines - part 2: the practical application of a new model Pharmacoepidemiol Drug Saf 2007 16 Suppl 1 S16 S41 17546574 10.1002/pds.1434

[B18] Singer DE Albers GW Dalen JE Fang MC Go AS Halperin JL Lip GY Manning WJ Antithrombotic therapy in atrial fibrillation: American College of Chest Physicians Evidence-Based Clinical Practice Guidelines (8th Edition) Chest 2008 133 6 Suppl 546S 592S 18574273 10.1378/chest.08-0678

[B19] Guyatt GH Sinclair J Cook DJ Glasziou P Users' guides to the medical literature: XVI. How to use a treatment recommendation. Evidence-Based Medicine Working Group and the Cochrane Applicability Methods Working Group JAMA 1999 281 19 1836 1843 10.1001/jama.281.19.1836 10340372

[B20] March JS Klee BJ Kremer CM Treatment benefit and the risk of suicidality in multicenter, randomized, controlled trials of sertraline in children and adolescents J Child Adolesc Psychopharmacol 2006 16 1–2 91 102 16553531 10.1089/cap.2006.16.91

[B21] Singh S Dolan JG Centor RM Optimal management of adults with pharyngitis–a multi-criteria decision analysis BMC Med Inform Decis Mak 2006 6 14 10.1186/1472-6947-6-14 16533386 PMC1431519

[B22] Elwyn G O'Connor A Stacey D Volk R Edwards A Coulter A Thomson R Barratt A Barry M Bernstein S Developing a quality criteria framework for patient decision aids: online international Delphi consensus process BMJ 2006 333 7565 417 10.1136/bmj.38926.629329.AE 16908462 PMC1553508

[B23] Boers M Brooks P Fries JF Simon LS Strand V Tugwell P A first step to assess harm and benefit in clinical trials in one scale J Clin Epidemiol 2010 63 6 627 632 10.1016/j.jclinepi.2009.07.002 19800197

[B24] Garrison LP Jr Towse A Bresnahan BW Assessing a structured, quantitative health outcomes approach to drug risk-benefit analysis Health Aff (Millwood) 2007 26 3 684 695 10.1377/hlthaff.26.3.684 17485745

[B25] Ponce RA Bartell SM Wong EY LaFlamme D Carrington C Lee RC Patrick DL Faustman EM Bolger M Use of quality-adjusted life year weights with dose–response models for public health decisions: a case study of the risks and benefits of fish consumption Risk Anal 2000 20 4 529 542 10.1111/0272-4332.204050 11051076

[B26] Dolan JG Shared decision-making–transferring research into practice: the Analytic Hierarchy Process (AHP) Patient Educ Couns 2008 73 3 418 425 10.1016/j.pec.2008.07.032 18760559 PMC2650240

[B27] Holden WL Juhaeri J Dai W Benefit-risk analysis: examples using quantitative methods Pharmacoepidemiol Drug Saf 2003 12 8 693 697 10.1002/pds.794 14762986

[B28] Sutton AJ Cooper NJ Abrams KR Lambert PC Jones DR A Bayesian approach to evaluating net clinical benefit allowed for parameter uncertainty J Clin Epidemiol 2005 58 1 26 40 10.1016/j.jclinepi.2004.03.015 15649668

[B29] Lynd LD O'Brien BJ Advances in risk-benefit evaluation using probabilistic simulation methods: an application to the prophylaxis of deep vein thrombosis J Clin Epidemiol 2004 57 8 795 803 10.1016/j.jclinepi.2003.12.012 15485731

[B30] Sherrill B Amonkar MM Stein S Walker M Geyer C Cameron D Q-TWiST analysis of lapatinib combined with capecitabine for the treatment of metastatic breast cancer Br J Cancer 2008 99 5 711 715 10.1038/sj.bjc.6604501 18728660 PMC2528149

[B31] Shakespeare TP Gebski VJ Veness MJ Simes J Improving interpretation of clinical studies by use of confidence levels, clinical significance curves, and risk-benefit contours Lancet 2001 357 9265 1349 1353 10.1016/S0140-6736(00)04522-0 11343760

[B32] Shaffer ML Watterberg KL Joint distribution approaches to simultaneously quantifying benefit and risk BMC Med Res Methodol 2006 6 48 10.1186/1471-2288-6-48 17038184 PMC1630697

[B33] Hauber AB Mohamed AF Johnson FR Falvey H Treatment preferences and medication adherence of people with Type 2 diabetes using oral glucose-lowering agents Diabet Med 2009 26 4 416 424 10.1111/j.1464-5491.2009.02696.x 19388973

[B34] Johnson FR Ozdemir S Mansfield C Hass S Miller DW Siegel CA Sands BE Crohn's disease patients' risk-benefit preferences: serious adverse event risks versus treatment efficacy Gastroenterology 2007 133 3 769 779 10.1053/j.gastro.2007.04.075 17628557

[B35] Johnson FR Ozdemir S Mansfield C Hass S Siegel CA Sands BE Are adult patients more tolerant of treatment risks than parents of juvenile patients? Risk Anal 2009 29 1 121 136 10.1111/j.1539-6924.2008.01135.x 18826414 PMC2847437

[B36] Chuang-Stein C A new proposal for benefit-less-risk analysis in clinical trials Control Clin Trials 1994 15 1 30 43 10.1016/0197-2456(94)90026-4 7908619

[B37] Osiri M Suarez-Almazor ME Wells GA Robinson V Tugwell P Number needed to treat (NNT): implication in rheumatology clinical practice Ann Rheum Dis 2003 62 4 316 321 10.1136/ard.62.4.316 12634229 PMC1754501

[B38] Johnson FR Van Houtven G Ozdemir S Hass S White J Francis G Miller DW Phillips JT Multiple sclerosis patients' benefit-risk preferences: serious adverse event risks versus treatment efficacy J Neurol 2009 256 4 554 562 10.1007/s00415-009-0084-2 19444531

[B39] Ryan M McIntosh E Shackley P Methodological issues in the application of conjoint analysis in health care Health Econ 1998 7 4 373 378 10.1002/(SICI)1099-1050(199806)7:4<373::AID-HEC348>3.0.CO;2-J 9683097

[B40] Edwards JE McQuay HJ Moore RA Collins SL Reporting of adverse effects in clinical trials should be improved: lessons from acute postoperative pain J Pain Symptom Manage 1999 18 6 427 437 10.1016/S0885-3924(99)00093-7 10641469

[B41] Lynd L Coombes M Rishidi A Sculpher M Willan A First steps in developing a risk-benefit analysis framework: a systematic review and critical evaluation of risk-benefit analysis methods 2004 York (UK): University of York http://www.york.ac.uk/media/che/documents/papers/presentations/04.pdf

[B42] Stang PE Pham SV Kinchen K Raff SB Mussen F Gondek K The identification of benefit in medical intervention: an overview and suggestions for process Am J Ther 2008 15 5 495 503 10.1097/MJT.0b013e31816b8fff 18806527

[B43] Puhan MA Chandra D Mosenifar Z Ries A Make B Hansel NN Wise RA Sciurba F The minimal important difference of exercise tests in severe COPD Eur Respir J 2011 37 4 784 790 10.1183/09031936.00063810 20693247 PMC5516638

[B44] Puhan MA Frey M Buchi S Schunemann HJ The minimal important difference of the hospital anxiety and depression scale in patients with chronic obstructive pulmonary disease Health Qual Life Outcomes 2008 6 46 10.1186/1477-7525-6-46 18597689 PMC2459149

